# Fascicle dynamics of the tibialis anterior muscle reflect whole-body walking economy

**DOI:** 10.1038/s41598-023-31501-2

**Published:** 2023-03-22

**Authors:** Samuel T. Kwak, Young-Hui Chang

**Affiliations:** School of Biological Sciences, Georgia Institute of Technology, Atlanta, GA USA

**Keywords:** Motor control, Biomedical engineering, Ultrasound, Musculoskeletal system

## Abstract

Humans can inherently adapt their gait pattern in a way that minimizes the metabolic cost of transport, or walking economy, within a few steps, which is faster than any known direct physiological sensor of metabolic energy. Instead, walking economy may be indirectly sensed through mechanoreceptors that correlate with the metabolic cost per step to make such gait adaptations. We tested whether velocity feedback from tibialis anterior (TA) muscle fascicles during the early stance phase of walking could potentially act to indirectly sense walking economy. As participants walked within a range of steady-state speeds and step frequencies, we observed that TA fascicles lengthen on almost every step. Moreover, the average peak fascicle velocity experienced during lengthening reflected the metabolic cost of transport of the given walking condition. We observed that the peak TA muscle activation occurred earlier than could be explained by a short latency reflex response. The activation of the TA muscle just prior to heel strike may serve as a prediction of the magnitude of the ground collision and the associated energy exchange. In this scenario, any unexpected length change experienced by the TA fascicle would serve as an error signal to the nervous system and provide additional information about energy lost per step. Our work helps provide a biomechanical framework to understand the possible neural mechanisms underlying the rapid optimization of walking economy.

## Introduction

It has been well established that humans prefer to walk with a gait pattern that minimizes their metabolic cost of transport (COT), which is a measure of energy used over a unit distance traveled. Maximizing the economy of walking has been observed when humans choose their preferred steady state walking speed^1,2^, stride frequency^3–5^, and step length^6^. When presented with a novel walking condition, humans can rapidly adapt to a near optimal walking pattern within only one or two steps^7^. Although whole body metabolic power (energy per unit time) may be measured directly by blood gas chemoreceptors^8,9^, no direct physiological sensor for whole body walking economy (i.e., metabolic COT, or energy per unit distance) has been identified. How then are humans able to control and optimize for a per step measure of whole-body energy utilization when it cannot be directly sensed?

In addition to directly measuring the local state of the proximate sensory organ, physiological sensors are also believed to indirectly contribute to the nervous system’s capacity to estimate more global, abstract variables that cannot be sensed by any single sensory organ. For example, muscle spindles provide a direct measurement of their own muscle fascicle lengths; however, they can in aggregate across multiple muscles also contribute to a centrally integrated estimation of a limb’s endpoint position in space^10,11^. There are other candidate sensors that could potentially contribute to such an abstract representation of whole-body metabolic COT. Use of a direct metabolic sensor, such as blood gas chemoreceptors, in conjunction with sensory organs that estimate distance walked may be one possible mechanism to estimate whole-body walking economy. While direct sensors of metabolic by-products may account for gait pattern selection over long time scales (minutes), blood gas chemoreceptors are ineffective at sensing metabolic changes on shorter time scales (seconds). The relatively high latency in blood gas chemoreceptors limit their ability to guide real-time adaptations that have been observed to minimize energy cost on a per-step basis^12^. Muscle proprioception may be a mechanism for rapid sensing of walking economy. Group III and IV afferent neurons are other potential candidates. These neurons are metabolically stimulated by muscle activity byproducts such as lactic acid to directly influence ventilation response^13–15^. However, they are also sensitive to mechanical stimulation by muscle contraction and stretch^14,16^. Feedback from group I and II afferent neurons provide rapid mechanical feedback on muscle function (millisecond timescale) and can be non-invasively inferred from measuring muscle fascicle dynamics via ultrasonography^17^. Currently, there is a paucity of evidence that proprioceptive feedback from individual muscles can map to walking economy. As a first step toward understanding the role of muscle proprioception in whole-body metabolic sensing during locomotion, we investigated whether muscle fascicles have the biomechanical capacity to be an indirect sensor of metabolic COT during human walking. Indeed, if the necessary mechanics of the muscle fascicles are not present, then the afferent neurons monitoring fascicle dynamics could not relay any useful information to the nervous system.

Muscle proprioception is primarily accomplished through the sensing of muscle length, velocity, and force. Muscle spindles are traditionally thought to sense static muscle length and changes in muscle length (i.e., fascicle velocity). Golgi tendon organs are known to sense muscle force^18,19^, although recent studies have suggested that spindles may also be correlated with a muscle’s force and yank^20,21^. Regardless of these details, muscle proprioceptive feedback generally provides a rapid estimate of the mechanical state of the muscle. It is prohibitively difficult to measure neural activity directly from these sensory neurons during human walking. In contrast, it is tenable to measure the biomechanical parameters such as muscle fascicle length and velocity, to which these afferent firing rates are known to be closely related during behavior^17^. Our rationale was that knowing the biomechanical state of the muscle fascicles during walking would provide important insights into what sensory feedback signals are available to the nervous system for estimating walking economy.

Lower limb proprioception is suggested to play an important role in metabolically-driven gait adaptation. Hubbuch et al.^22^ demonstrated a delay in adaptation to a more economical gait from level to uphill walking when applying a noisy vibration to the Achilles tendon to disrupt muscle spindle feedback. Increased eccentric lengthening occurs along the triceps surae muscle when suddenly switching from level to uphill walking, and Achilles tendon vibration, which selectively activates muscle spindle feedback^23,24^, would inhibit the ability to accurately sense muscle length changes. It remains unclear, however, whether proprioception of muscle fascicle dynamics can contribute to a greater sensory representation of whole-body walking economy. As a starting point to gain a better understanding of how proprioception influences gait economy, we aimed to characterize the relationship between muscle fascicle dynamics in a candidate leg muscle over a range of walking conditions designed to vary whole body COT.

Though information from many muscles throughout the gait cycle may likely contribute, we chose to focus on testing a single candidate muscle, the tibialis anterior (TA), that was likely to demonstrate a relationship between muscle fascicle length dynamics and metabolic COT. The U-shaped COT curve commonly observed across different walking speeds and frequencies arises partly from the economical, pendulum-like transfer from gravitational potential energy to forward kinetic energy. COT is minimized at the speed when this transfer is maximized^25^. During this pendular exchange, energy in the vertical direction is redirected to the forward direction at the beginning of the early stance phase of walking when the potential energy is at its minimum and the kinetic energy is at its maximum^26^. If there were an indirect sensor for COT based on mechanical energy exchange, the beginning of the early stance phase appears to be the ideal time to receive feedback. If non-optimal gait mechanics were adopted resulting in an inefficient energy exchange, it should manifest during the early stance phase of walking. Prior work has shown that the negative work that occurs during this early stance phase correlates with the metabolic cost of walking with a substantial amount of the work occurring at the ankle^27–29^. During split-belt walking adaptations, which are believed to be driven by minimizing metabolic cost^30^, TA muscle activation along with the anterior braking force during the early stance phase exhibit adaptive behavior while the posterior propulsive force during late stance does not^31^. The TA muscle also serves an important role in determining the walk-to-run transition speed (WRT), which is influenced at least in part by metabolic cost^4^. When wearing an ankle exoskeleton that provides an assistive plantarflexion torque during early stance phase, the increased ankle plantarflexion velocity leads to increased TA muscle activity along with a lower WRT^32^. These findings support the argument that ankle angular velocity and TA activation in early stance plays a role in determining the WRT^33^ and may contribute to the control of gait. Therefore, the TA muscle represents a tractable testbed for understanding the role that muscle proprioception could play in estimating the metabolic COT. We rationalized that this relationship would be apparent in TA muscle fascicle dynamics across different walking conditions that span the metabolic COT parameter space.

We hypothesized that TA fascicle lengthening velocity during the early stance phase of walking would reflect whole-body walking economy. Using established paradigms for manipulating the whole-body metabolic COT during walking, we systematically varied walking speed and step frequency as we measured resulting TA muscle fascicle dynamics. Recent studies have suggested that, on average over many steps, TA muscle fascicles remain isometric over the early stance phase^34,35^, which would obviate the ability to sense economy via muscle lengthening. We first tested whether TA muscle fascicles were isometric on individual steps during the early stance phase of gait. Secondly, we tested if peak TA fascicle lengthening velocity during early stance phase would correlate with whole-body COT.

## Results

**Figure 1:**
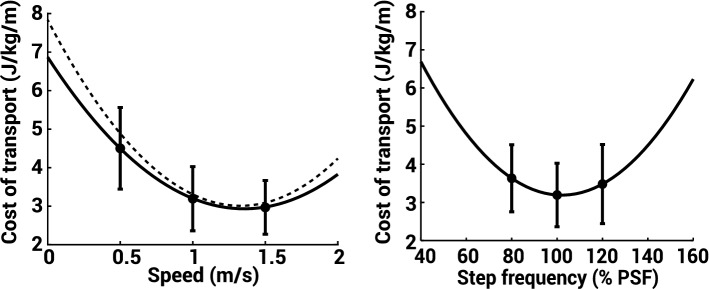
COT landscape curves with respect to walking speed (left) and step frequency (right). Data shown are means (filled circles) and standard deviations (error bars) (n = 10). The solid curves show a 2nd order polynimial fit of the mean data. The dashed curve shows the COT vs speed equation derived by Browning and Kram^2^.

Changes in the metabolic COT resulting from both walking speed and step frequency changes across subjects followed a U-shaped curve relationship (Fig. 1). Particularly for speed changes, our results agreed with the relationship predicted by Browning and Kram^2^ (R^2^ = 0.99).

**Figure 2:**
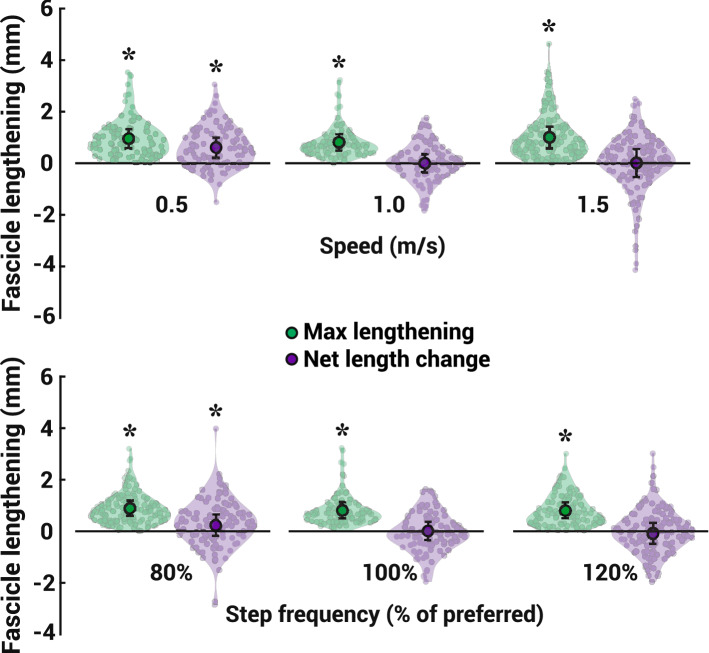
TA fascicle length changes during early stance across different walking speeds (top) and step frequencies (bottom). Mean values with standard deviation bars are superimposed on violin plots representing the maximum fascicle lengthening (green) and the net change in fascicle length (purple) observed for every step of every subject. Asterisks above violin plots indicate significantly greater than zero (α < 0.05).

Nearly all individual steps analyzed exhibited some degree of fascicle length change in the TA muscle during early stance. Fascicle lengthening occurred in 96.7 ± 1.5% of all steps analyzed across all subjects and conditions. All conditions exhibited a significant amount of maximum lengthening over the early stance phase (1.0 ± 0.7, 0.9 ± 0.6, 0.8 ± 0.6, 0.8 ± 0.6, 1.0 ± 0.8 mm for the 0.5 m/s, 80% PSF, 1.0 m/s at 100% PSF, 120% PSF, and 1.5 m/s gait conditions, respectively), which we defined as the greatest amount of continuous lengthening in the window from heel contact to foot-flat. Despite these observations, we observed little net length change over the entire early stance phase (0.6 ± 0.8, 0.2 ± 0.8, 0.0 ± 0.7, − 0.1 ± 0.8, 0.0 ± 1.1 mm for the 0.5 m/s, 80% PSF, 1.0 m/s at 100% PSF, 120% PSF, and 1.5 m/s gait conditions, respectively), which we defined as the net difference in fascicle length at heel contact compared to foot-flat. The mean net length change of the TA muscle fascicles did not significantly change during steps when walking at 1.0 m/s at PSF (*t*(7) = 0.32, *p* = 0.319), 1.5 m/s (*t*(10) = 0.03, *p* = 0.974), 80% PSF (*t*(10) = 1.74, *p* = 0.112), and 120% PSF conditions (*t*(9) = − 1.21, *p* = 0.256). We observed a small but significant net length change when subjects walked at 0.5 m/s (*t*(8) = 2.93, *p* = 0.019) (Fig. 2).

**Figure 3:**
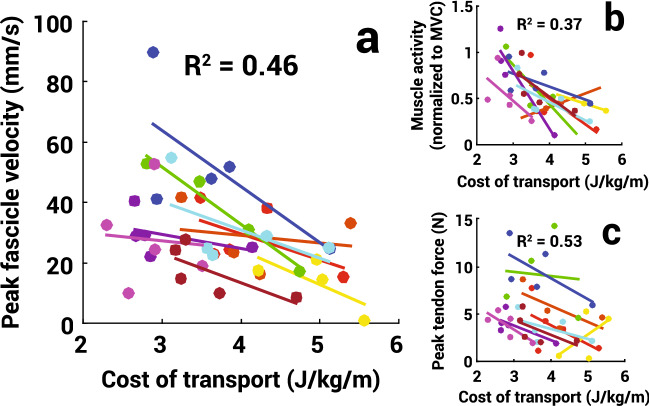
(**a**) Peak TA fascicle lengthening velocity, (**b**) peak TA muscle activity, and (**c**) peak TA tendon force as a function of whole-body metabolic COT with the adjusted R^2^ for the linear mixed model across all subjects. Each color represents an individual subject with dots as the mean value for a given walking condition and lines as the linear fit for a single subject across conditions.

In all subjects, peak lengthening velocity of the TA fascicles decreased proportionally as the whole-body metabolic COT increased (*t*(40) = − 3.80, *p* < 0.001, R^2^ = 0.46, Fig. 3a). The mean velocities for the 0.5 m/s, 80% PSF, 1.0 m/s at 100% PSF, 120% PSF, and 1.5 m/s gait conditions were 24.7 ± 13.1, 32.0 ± 11.6, 29.8 ± 9.4, 29.5 ± 17.6, and 31.4 ± 16.8 mm/s, respectively. For those same gait conditions, the mean COT values were 4.5 ± 1.1, 3.6 ± 0.9, 3.2 ± 0.8, 3.5 ± 1.0, and 3.0 ± 0.7 J/kg/m, respectively. To better understand how this relationship arose, we looked at two contributing factors to TA muscle fascicle velocity, TA peak muscle activity and TA peak tendon force, and found that both factors had a significant negative correlation with COT (muscle activity: *t*(34) = − 4.76, *p* < 0.001, R^2^ = 0.37; tendon force:* t*(40) = − 2.55, *p* = 0.015, R^2^ = 0.53, Fig. 3b,c). We also observed that torque and angular velocity about the ankle joint were both significantly correlated with peak fascicle velocity (ankle torque: *t*(44) = 3.03, *p* = 0.004, R^2^ = 0.40; angular velocity:* t*(44) = 2.60, *p* = 0.127, R^2^ = 0.37).

**Figure 4:**
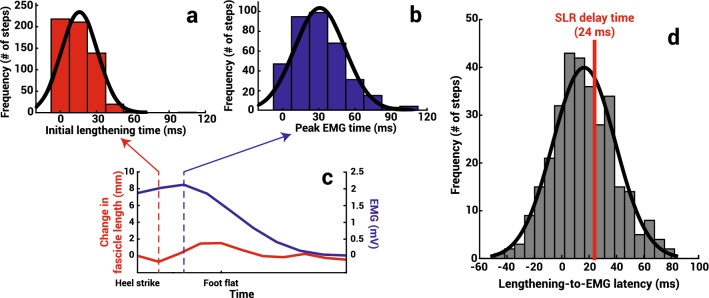
Histograms with normal distribution curves for the times after heel contact of the (**a**) initial TA fascicle lengthening (red) and (**b**) peak TA EMG activity (blue). (**c**) The TA fascicle length change (red) and relative EMG (blue) of the TA muscle from a representative step cycle with arrows indicating when initial lengthening (red arrow) and peak EMG (blue arrow) occur. Only a partial gait cycle is shown here. (**d**) Histogram with a normal distribution curve for the latency from initial lengthening to peak EMG with an indication (red) of the earliest time a short latency response (SLR) would occur.

Across all steps analyzed over all trials, the initial lengthening of the TA fascicle, when the fascicle first lengthens after heel strike, began on average at 16.0 ± 15.1 ms after heel contact, while peak TA EMG activity occurred 31.2 ± 20.9 ms after heel contact (Fig. 4). The average difference between this EMG activity and the initial lengthening was significantly less than the predicted short latency response of 24 ms^36^ (*M* ± *SD* = 16.18 ± 22.99 ms, *t*(321) = − 6.11, *p* < 0.001).

## Discussion

In this study, we investigated how tibialis anterior muscle fascicle dynamics correlated with walking economy. In doing so, we found the peak velocity of those muscle fascicles as they were being lengthened during the earliest portion of stance phase inversely correlates with the whole-body metabolic COT. Muscle activation timing suggests that the TA muscle is activated in anticipation of the collision and resultant stretch rather than as a reflex response and may be a means of predicting the amount of stretch that will be experienced by the muscle. Therefore, proprioceptive feedback from the TA muscle in early stance may function more as a means to estimate the error from the anticipated stretch rather than to directly regulate the stretch. These biomechanical observations of in vivo muscle function provide plausible evidence that proprioceptive feedback from the TA muscle during only a small portion of a single step has the capacity to act as a rapid indirect sensor for whole body walking economy.

When considering potential physiological sensors and gait events in which whole-body walking energetics can be estimated, the TA muscle during early stance is an appealing candidate. Energy lost during the collision, which should correspond to the energy used during propulsion for steady walking^37^, is closely linked to whole-body energy expenditure^27^. The early stance phase of the gait cycle is when this dorsiflexor muscle displays maximum muscle activation to control ankle plantarflexion from heel strike to when the foot falls flat on the ground. Moreover, our results show that TA muscle activity during early stance correlates to walking economy. The metabolic power required for TA muscle activation in early stance is relatively small compared to other muscles over other phases of the gait cycle. Furthermore, TA activation is independent of the propulsive force required of the ankle extensor muscles that typically power walking^38^. This allows sensory sampling by the TA muscle to remain independent from the muscular effort that determines whole-body energy costs, which may be particularly important for sensing walking economy at slower walking speeds where propulsive requirements are low. As we have shown, whole-body metabolic COT reflects TA muscle fascicle velocity, which is closely related to muscle spindle sensory feedback^17^. Although other muscles may be contributing, the TA muscle may be particularly suitable for estimating whole-body walking economy during the early stance phase of the gait cycle.

Our results show that muscle proprioceptive feedback correlate well with more complex, abstract variables beyond the proximate muscle length dynamics. Spindle function has traditionally been thought of as limited to proprioception and stretch-evoked muscle activity^39^ and is often seen as a simple transformation of the local fascicle length and velocity into spindle firing rate^17,19^. Gamma motor drive of intrinsic muscle fibers can alter the sensitivity of muscle spindles^40^, allowing a muscle spindle to tune its feedback in a task-dependent manner that is related to the descending motor command. Moreover, muscle spindle firing rate more closely predicts future muscle velocity than it does present muscle velocity^41^. In this framework, the gamma motor drive to TA muscle fibers can be seen as a prediction of the magnitude of the collision at heel-strike. Though the spindles more directly sense muscle stretch, this stretch arises during gait from the imposed force on that muscle in early stance and the activation of that muscle to stiffen the ankle joint in anticipation of the collision with the ground. Muscle spindles, and consequently muscle stretch, can thus provide feedback about the prediction made regarding the collision in early stance phase. Indeed, we observed that there was a strong correlation between TA fascicle lengthening and both ankle torque and ankle angular velocity, indicating that the joint mechanics associated with early stance phase directly transferred to TA fascicle dynamics.

The force experienced by the lead leg in early stance phase reflects both the downward vertical acceleration of the body due to gravity and the forward velocity generated by the propulsion of the trailing contralateral leg. The maximum forward and downward center of mass velocities occur in the period between heel contact and foot-flat^26^. Therefore, it seems reasonable to suggest that feedback from muscle stretch in the early stance phase of walking can relay information regarding the gravitational potential and forward kinetic energies exchanged during walking, a factor that has been tied closely with locomotor economy^25,26^. It would therefore be reasonable that stretch experienced by the muscle fascicles in early stance could serve as a rapid and immediate estimation of the energy cost of walking and could be used as error-based feedback for making corrections on subsequent steps.

When walking at or faster than the preferred walking speed, the locomotor economy increases with speed along with the increased need for energy absorption at the ankle during the collision^27^. Additionally, TA muscle activation and TA tendon stretch, which is related to TA tendon force, also increase^35^. As we studied walking at or slower than the preferred speed, we saw the opposite effect. This nonlinear, U-shaped relationship in which COT decreases then increases as a variable continually increases in value has been observed with walking speed^1,2^, stride frequency^3,5^, and TA muscle activity^42^. Prior studies have shown that muscle proprioceptive feedback is best modeled as a non-linear system^21,43–45^. So even as the amount of energy absorbed through muscle fascicle stretch may continually increase with walking speed, its relationship does not necessarily need to be linear to be useful to the nervous system. We were limited in this study to only testing lower walking speeds to accommodate the practical ranges of the different step frequency and step length requirements of our walking conditions. It will be important for future work to study these muscle fascicle dynamics at faster speeds to confirm a more comprehensive picture of this potential relationship.

Though the TA muscle is an appealing candidate to test whether muscle proprioception can estimate whole-body COT, we suspect it may be one of many muscles that are functioning in this way. It is likely that other lower limb muscles may be contributing to this rapid estimation to capture a more complete picture of the whole-body energy cost per step. Since we looked at the TA unilaterally, the contralateral TA muscle would also contribute to proprioceptive feedback to estimate COT. Other ankle dorsiflexor muscles such as the extensor digitorum longus act in parallel with the TA and could provide additional feedback. Knee extensors and hip extensor muscles also likely undergo muscle lengthening during the early stance collision and could be used to help improve the accuracy of walking economy estimates. Aside from these muscles which absorb energy during the early stance phase, feedback can also be obtained from propulsive muscles that generate energy as well as muscles involved in the swing phase of walking^46–48^. In the case of these muscles, it is believed that their ability to sense energy relates directly to the amount of muscle activity serving more as an effort sensor rather than an energy exchange sensor^48^. Sensing effort with propulsive muscles may be effective at fast walking speeds when COT increases directly with effort. However, a more complex sensing model would be needed at slow speeds where COT is observed to now increase with less effort from propulsive muscles. Although we suspect that integrated proprioceptive feedback from several lower limb muscles may provide a rapid and accurate means of representing walking economy, more work is needed to build a more comprehensive understanding of how the nervous system rapidly models and predicts whole body energetics.

Our hypothesis of the proprioceptive importance of fascicle dynamics to estimating walking economy depends upon the existence of fascicle lengthening during the early stance phase. Ours is the first study to report that the TA muscle fascicles almost always experience some degree of length change on every step within the period between heel strike and foot-flat. During this portion of the walking gait cycle, the muscle tendon unit necessarily lengthens due to the highly stereotyped ankle plantarflexion that occurs immediately after heel strike. Yet, previous studies measuring in vivo TA fascicle dynamics during human walking have reported that the TA muscle fascicles, on average, behave isometrically during this period^34,35^. These studies concluded that this functionally allows energy storage and return within the TA tendon while also protecting the TA muscle from injurious fascicle strain. But, when considering the fascicle dynamics on individual steps, our results reveal that high variability in the timing of fascicle dynamics can result in the averaged behavior over many steps to appear isometric. There are other methodological differences in our work that may help to explain perceived disparities to previous work. For example, we collected our ultrasound data at 60 Hz, or about once every 1.7% of the gait cycle, while Chleboun and colleagues^34^ measured fascicle length at only seven points throughout the entire gait cycle with 0, 7, and 46% being the measurements closest to the early stance period we focused on. This lower sampling rate would make it more likely to miss brief periods of fascicle length change within the step cycle. Although we measured a small delay of < 1 frame in our ultrasound data relative to our kinematics data, it would not affect our general conclusions made from our results because even with a compensatory ultrasound delay, the majority of observed time differences between fascicle stretch and peak EMG would still be below the minimum latency to be considered a stretch reflex response. Though not reported in their work, Maharaj and colleagues^35^ also acknowledged observing notable shortening and lengthening peaks in their individual fascicle velocity measurements. Moreover, our findings agree with a musculoskeletal model of the TA muscle during gait indicating that TA fascicle lengthening should occur in early stance phase^49^. While we showed TA fascicles experience some form of lengthening during the early stance phase of nearly every step taken, this does not contradict the suggestion that the TA tendon lengthens much more than the muscle and may play a significant role in energy dissipation or injury prevention. It is worth noting that we observed fascicle length changes on the millimeter scale compared to the centimeter scale of MTU length changes which is used in other studies^34,35^. However, muscle spindles are known to be sensitive and provide meaningful information for fascicle length changes in the sub-millimeter range^17,50^, which is well within our observed data.

While we were able to show that the peak velocity of TA muscle fascicle lengthening correlates with whole-body energetics when averaged across a walking trial, we also observed noticeable step-to-step variability in the amount of lengthening experienced by the fascicles. Though some degree of fascicle lengthening was observed in almost every step across all subjects and conditions, the amount of lengthening varied even on sequential steps within the same trial for a single subject. Even in steady-state conditions, humans have an inherent amount of gait variability, which may reflect the natural motor abundance of the locomotor system^51–53^. It is possible that such natural variability in fascicle length change could be detrimental to a system with a low signal-to-noise ratio. Our results in steady-state conditions open the possibility that the locomotor system may be able to distinguish relevant differences in fascicle stretch above some noise threshold. Although our subjects always walked under steady-state conditions within a given trial, we varied our walking conditions across trials enough to emulate the significant step-to-step perturbations that might cause an energetically-driven gait adaptation. This points to a general limitation of experimentally measuring whole-body energetics with indirect calorimetry, which can only be done over several minutes and numerous steps. This limitation in rapidly measuring whole-body energetics, however, is not different from the challenge that the nervous system has in trying to estimate walking economy on a given step. We rationalized that the correlation of COT with average peak fascicle velocity observed during steady-state walking would reflect a similar relationship on any single step.

**Figure 5:**
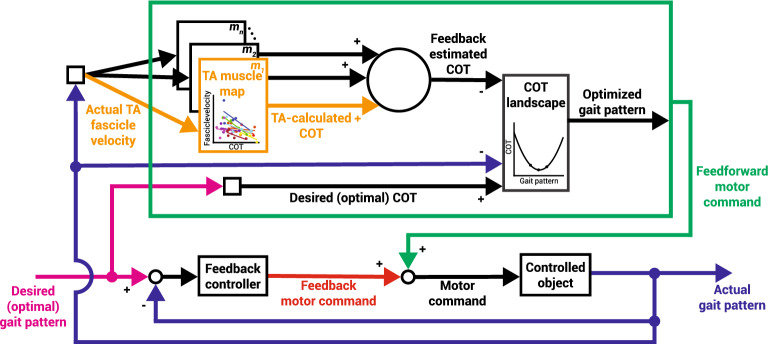
Proposed feedback control model of gait using cost of transport (adapted from Wolpert et al.^54^). The controlled object, which is a physical entity controlled by the central nervous system such as the legs, consists of transformations from motor command to the motion of the controlled object resulting in a gait pattern. “Optimal” can be considered the gait pattern that minimizes the COT. The actual gait pattern manifests itself through the lengthening and shortening of a subset of *n* muscles. Each respective muscle map *m*_*n*_ correlates individual muscle feedback and whole-body COT, such as TA muscle fascicle velocity as derived from our study (orange, Fig. 3). The individual muscle estimates can be summed to create a more accurate representation of whole-body COT. This feedback-estimated COT value along with the actual gait pattern provides a state estimation within the COT landscape (Fig. 1). An error-based correction can then be made to ultimately generate a predicted optimized gait pattern as a feedforward motor command.

So how can rapid feedback from muscle dynamics be used to alter one’s gait? As a first step, we propose applying a commonly accepted feedback-error-learning model^54^ as a framework for understanding the role of muscle proprioception in optimizing gait economy (Fig. 5). This sort of control adaptation framework has been observed in split-belt walking^31,55^, hopping^56^, and hand-movement tasks^57,58^, among other movements. In this proposed model, the gait pattern can be seen as relating to any combination of high-level parameters such as walking speed, step frequency, or step width which result from a specific muscle activation pattern. As an initial motor command is sent to the muscles, proprioceptive feedback related to muscle stretch would be converted to the COT dimension via a previously learned relationship between the two. Whether this gait pattern is optimal is dependent on where the chosen gait pattern sits within the COT landscape. As people adjust their gait pattern, this would allow for greater knowledge of the COT landscape to better inform if they are walking at their energetic optimum^5^. Here, we specifically explored the feasibility of one important part of this proposed model. We characterized the muscle-COT relationship for the tibialis anterior muscle under different walking conditions. This indicated the possible role that muscle proprioceptive feedback plays during rapid gait optimization.

The ability to adjust quickly and economically to changing conditions is an important determinant of human locomotor adaptation. Yet, we currently do not have a good understanding of how the nervous system can rapidly sense whole body walking economy to drive these adjustments. In this study, we demonstrate that the peak fascicle lengthening velocity from a single muscle over a very brief time window has the potential to reflect the whole-body metabolic COT when walking with different gait speeds and step frequencies. It is reasonable to infer that any number of additional proprioceptive inputs from multiple lower limb muscles may be integrated to make accurate predictions of walking economy within only one or two walking steps. This provides a physiologically viable explanation for how rapid, step-by-step adjustments are made by the nervous system to quickly optimize walking economy under novel conditions.

## Methods

### Experimental protocol

**Figure 6:**
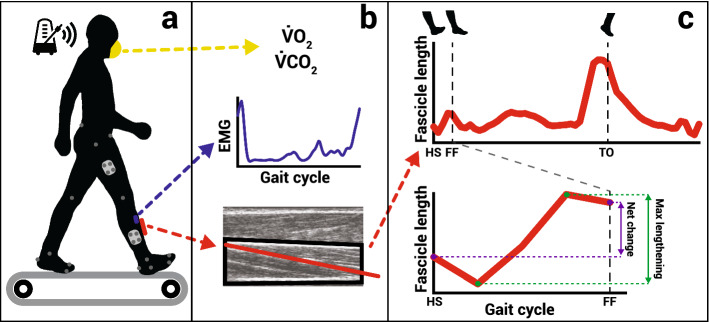
(**a**) Subjects walked to a metronome beat on a treadmill at a set speed. (**b**) We measured whole body indirect calorimetry (yellow) as well as EMG (blue) and B-mode ultrasound (red) from the TA muscle during walking. (**c**) We determined gait events such as heel strike (HS), foot-flat (FF), and toe-off (TO) using retroreflective markers and ground reaction force measurement. We used the ultrasound images to measure TA muscle fascicle length across the gait cycle (top). We calculated the net length change (purple) and single maximum lengthening episode (green) of the fascicle during the period between heel strike and foot-flat (bottom).

We recruited 11 healthy adults (6 males; age: 22.8 ± 3.6 years; body mass: 64.4 ± 9.7 kg) with no major musculoskeletal or cardiovascular abnormalities. All participants gave written, informed consent prior to participating in the protocol approved by the Georgia Institute of Technology Institutional Review Board. All methods in this study were performed in accordance with this approved protocol. Subjects walked on a treadmill in two sets of 5 different conditions in a pseudo-randomized order, once for the ultrasound, electromyography (EMG), and motion analysis collection and once for the metabolic data collection, totaling 10 walking trials (Fig. 6). In three of those walking conditions, we altered walking speed between a slow, medium, and fast speed (0.5, 1.0, and 1.5 m/s, respectively). Due to unexpected technical issues, not all conditions were collected on all subjects. In these cases, sample sizes are indicated accordingly. Subjects were allowed one minute to walk at a comfortable step frequency at each of those speeds before a preferred step frequency (PSF) was determined. After this, a metronome was set at that PSF, and subjects were asked to match their step frequency to the metronome for the duration of the remaining trials. For the remaining two conditions, subjects walked at 1.0 m/s while matching their step frequency to the beat of a metronome set to a low (80% PSF) or high (120% PSF) frequency. During preliminary testing, we found this frequency range to be the limits in which all subjects we able to perform at 1.0 m/s.

### External kinematics and kinetics

During the ultrasound collection, subjects walked on a custom dual-belt instrumented treadmill (1080 Hz). We simultaneously measured kinematic data with an eight-camera motion analysis system (120 Hz, Vicon Motion Systems, Oxford, UK) with attached retroreflective markers on the pelvis and lower extremities of each subject. 3-D motion and force measurements were processed through biomechanics analysis software (Visual3D, C-Motion, Germantown, MD, USA), where we used the ground reaction force and ankle joint angle to determine heel contact and foot-flat events, respectively. All kinetic and kinematic variables were calculated in the sagittal plane.

### Electromyography

We used surface electrodes to record EMG data (1080 Hz) from the same TA muscle on which the ultrasound probe was placed. We then lowpass filtered using a Butterworth filter with a cutoff frequency of 433 Hz, rectified, and smoothed the EMG data using a 100 ms moving average. The EMG data were normalized to each subject’s maximum activation recorded during the experiment.

### Indirect calorimetry

To measure metabolic power, we used an indirect calorimetry system to measure rates of oxygen and carbon dioxide exchange (ParvoMedics TrueOne 2400, Salt Lake City, UT, USA). Subjects walked on a treadmill for 6 min at each condition and we averaged the final 3 min from each trial to measure the steady state volume rates of oxygen consumption and carbon dioxide production for that trial condition. We then calculated the gross metabolic rate using the Brockway equation^59^ and normalized it to each subject’s body mass. We further normalized the gross metabolic rate to the walking speed to calculate walking economy, or the metabolic COT (J/kg/m)^2^.

### Muscle dynamics calculations

We imaged TA muscle fascicles at 60 Hz using B-mode ultrasound through a 96-element linear array probe (Echoblaster 128, Telemed, Vilnius, LTU) at a frequency of 6 MHz with a field of view of 65 mm using ultrasound scanning software (Echo Wave II, Telemed, Vilnius, LTU). We placed the probe at the mid-belly of the muscle as the TA was scanned perpendicular to the surface of the skin before being wrapped to secure the probe in place. If the aponeuroses of the TA muscle were not parallel to one another in the ultrasound image, we adjusted the angle of the probe axially and placed modeling clay along the sides of the probe before wrapping to maintain the proper angle. During data collections, the ultrasound scanner sent a syncing trigger signal to the motion analysis system so that we would be able to determine when heel contact and foot-flat gait events occurred with respect to the ultrasound data.

We used a semi-automated tracking algorithm^60^ with manual correction to measure the fascicle length across the entire gait cycle (Supplementary Information). In short, this involved first identifying the deep compartment of the TA muscle, bounded by the middle and deep aponeuroses. We chose to analyze the deep compartment rather than the superficial because those fascicles were more easily visible across subjects. We measured the fascicle length by placing digital markers on the aponeuroses at the endpoints of a chosen muscle fascicle. The tracking algorithm, which implements an affine optic flow model, would then predict the position of the endpoints for the remainder of the frames. When the algorithm made a visibly incorrect prediction, we manually repositioned marker in the correct position. If one of the markers went out of the frame of the image, the tracking software allowed for the marker to be placed out of frame at the inferred position based on extrapolation of the aponeuroses.

We calculated fascicle velocity by taking the time derivative of these tracked fascicle length measurements. We defined peak fascicle velocity as the maximum lengthening velocity value between heel contact and foot-flat. We defined the net length change as the difference in fascicle length values when measured at heel strike and foot-flat and maximum lengthening as the greatest continuous length change over the same period (Fig. 6c). The initial lengthening of the fascicle was defined as the first frame after heel strike in which lengthening was observed. We calculated TA tendon force by dividing the ankle moment by the TA tendon moment arm length. The TA tendon moment arm was determined placing a marker on the TA tendon where it passed under the inferior extensor retinaculum and calculating the distance between that marker and the ankle joint center as determined by the biomechanics analysis software^61^.

### Statistical analysis

We filtered all raw kinematics and force plate data through a lowpass Butterworth filter with a cutoff frequency set at 6 Hz. We generated COT curves with respect to walking speed and step frequency by fitting a 2nd order polynomial curve onto the mean data and determining fit with a regression analysis. We tested if the net length change and the maximum lengthening observed across all steps were significantly greater than zero by conducting a one sample, one-tailed t-test. Muscle fascicle velocity data were excluded when there were less than three data points available to create a linear fit. To compare the peak fascicle lengthening velocity to the metabolic COT, we fitted the data to a linear mixed effect model with the subject as a random effect (Fascicle Velocity ~ COT + (1 | Subject)) and calculated an adjusted Pearson’s correlation coefficient for each subject. We replaced the fascicle velocity term with peak TA EMG and peak TA tendon force to determine how much those terms reflect the COT. We also replaced the COT term with peak ankle velocity and peak ankle torque to determine if greater stretch occurs due to the force or angular velocity acting about the ankle during the plantarflexion. All linear mixed effect models were followed by a two-sided t-test on the fixed effects to determine the significance of the fit. We also measured the difference between the timing of the peak TA muscle activity after heel strike and initial lengthening of the fascicle within the same step and used a one-sided one-sample t-test to see if the latency was less than that of an expected short latency stretch reflex (24 ms)^36^. All peak values were taken between heel contact and foot-flat, and all statistical analyses were performed with a significance threshold α = 0.05.

## Supplementary Information

Supplementary Video 1.

Supplementary Legends.

## Data Availability

The dataset used in this study is available from the corresponding author on reasonable request.
