# Supplementary material for: Fascicle dynamics of the tibialis anterior muscle reflect whole-body walking economy
Source: Sci Rep. 2023 Mar 22;13:4660. doi: 10.1038/s41598-023-31501-2 (PMC10033896; doi:10.1038/s41598-023-31501-2)
Supplement: Supplementary file 2 — Supplementary Legends. [file 41598_2023_31501_MOESM2_ESM.docx]

Supplementary Video 1. Ultrasound video of the tibialis anterior muscle during the early stance phase with muscle fascicle tracking in the deep compartment. Examples are given in which a fast (left) and slow (right) peak fascicle velocity is observed for the same subject walking under the same gait condition. The video is slowed down, looped, and oriented so that the distal end of the TA muscle is on the left side.
